# An interaction-based drug discovery screen explains known SARS-CoV-2 inhibitors and predicts new compound scaffolds

**DOI:** 10.1038/s41598-023-35671-x

**Published:** 2023-06-06

**Authors:** Philipp Schake, Klevia Dishnica, Florian Kaiser, Christoph Leberecht, V. Joachim Haupt, Michael Schroeder

**Affiliations:** 1 Bioinformatics, Biotechnology Center (BIOTEC), Technische Universität Dresden, Dresden, Saxony Germany; 2 University of Verona, Verona, Italy; 3 PharmAi, Dresden, Germany

**Keywords:** Virtual screening, Virtual drug screening, Respiratory tract diseases, Data acquisition

## Abstract

The recent outbreak of the COVID-19 pandemic caused by severe acute respiratory syndrome-Coronavirus-2 (SARS-CoV-2) has shown the necessity for fast and broad drug discovery methods to enable us to react quickly to novel and highly infectious diseases. A well-known SARS-CoV-2 target is the viral main 3-chymotrypsin-like cysteine protease (M^pro^), known to control coronavirus replication, which is essential for the viral life cycle. Here, we applied an interaction-based drug repositioning algorithm on all protein-compound complexes available in the protein database (PDB) to identify M^pro^ inhibitors and potential novel compound scaffolds against SARS-CoV-2. The screen revealed a heterogeneous set of 692 potential M^pro^ inhibitors containing known ones such as Dasatinib, Amodiaquine, and Flavin mononucleotide, as well as so far untested chemical scaffolds. In a follow-up evaluation, we used publicly available data published almost two years after the screen to validate our results. In total, we are able to validate 17% of the top 100 predictions with publicly available data and can furthermore show that predicted compounds do cover scaffolds that are yet not associated with M^pro^. Finally, we detected a potentially important binding pattern consisting of 3 hydrogen bonds with hydrogen donors of an oxyanion hole within the active side of M^pro^. Overall, these results give hope that we will be better prepared for future pandemics and that drug development will become more efficient in the upcoming years.

## Introduction

The COVID-19 pandemic, which started in Wuhan (China) and then spread worldwide, has caused almost 609 million infections and more than 6 million deaths as of September 2022 (World Health Organization). Its causative agent the Severe Acute Respiratory Syndrome Coronavirus 2 (SARS-CoV-2) belongs to the Coronaviridae family of single-stranded positive-sense RNA viruses^1,2^. Other viruses of the same family, namely the Severe Acute Respiratory Syndrome Coronavirus (SARS-CoV) and the Middle East Respiratory Syndrome coronavirus (MERS-CoV)^3^ already led to epidemics in 2002/3 and 2012 respectively^4,5^. Due to the severity of the current outbreak, the scientific community has undergone huge efforts to experimentally determine SARS-CoV-2 genome sequences and three-dimensional structures as fast as possible. The unseen amount of publicly available data on a single virus is the groundwork for developing virus-specific drugs that could end the current pandemic. The SARS-CoV-2 genome encodes for structural proteins and non-structural proteins such as 3CL^pro^, PL^pro^, helicase, and RNA-dependent RNA polymerase^6^. The four non-structural proteins mentioned above are key enzymes in the viral cycle^7^.

The Main protease (M^pro^) is being studied a lot in terms of structural and functional properties because of its high similarity, with significant conservation in the cleavage site, shared with SARS-CoV^8^. It is an enzyme involved in the processing of polyprotein which is translated from viral RNA^9^. Therefore, the inhibition of M^pro^ would ultimately suppress viral replication. Furthermore, there are no human proteases with a similar cleavage specificity as M^pro^, making it very unlikely for M^pro^ inhibitors to be toxic^10^. Considering this evidence, we will put the main effort into the SARS-CoV-2 target M^pro^.

In general, there are two main groups of methods that aim to identify new drugs for a given target, such as M^pro^, which are computational and experimental approaches^11^.

The wide range of in vitro experimental approaches performed to manage the pandemic includes studies aiming to determine appropriate drug targets^12^, newly developed experimental methods to validate predicted drugs^13–15^, experiments to uncover drug mechanisms^16–18^, and high throughput drug repurposing experiments^19^. One of the most important outcomes of experimental approaches is the development of the by-now-approved drug Paxlovid, a combination of nirmatrelvir^20^ and ritonavir, for treating COVID-19 patients with a very high risk of severe illness^21^. Furthermore, Boceprevir and GC-376 are identified as potent SARS-CoV-2 main protease inhibitors^22^. Nevertheless, experimental approaches in drug discovery require a high level of training, are expensive, and are generally less suited to perform large throughput studies to evaluate extensive compound libraries^23^. The above-mentioned drug Paxlovid for example is a derivative of a drug that was already developed as a potential SARS-CoV-1 inhibitor^20^.

Besides in vitro approaches aiming to identify potential new drugs, others are aiming to detect three-dimensional active site structures and compound binding modes. Structures obtained and published in the protein database (PDB) early on showed compound fragments in complex with M^pro^. They revealed the importance of the residues His41 and Cys145 that comprise the catalytic dyad similar to M^pro^ of SARS-CoV-1^24,25^. Further work disclosed that in M^pro^ an oxyanion hole is composed of Gly143, partly Ser144, and Cys145^10,26^ implying that a promising drug candidate should be able to interact covalently or noncovalently with at least one of these residues. However, these structures should be used with caution. It was shown that especially the M^pro^ structures generated with high-throughput methods are often lacking the representation of a possible important water molecule that could serve as a third catalytic residue and that the models are not on par with other structures in the PDB^27^. In addition, most structures are generated at temperatures of 100 K and thus are representing an active site configuration that is non-physiological, leading to errors such as the previously mentioned missing water molecule^28^. Nonetheless, structural approaches are extremely important to get insights into protein function and have already uncovered the mechanism of the FDA-approved SARS-CoV-2 inhibitor Remdesivir ^29^.

To cope with the problems of experimental approaches and to make use of the available data, computer-aided approaches in drug discovery are becoming more and more popular and important^11^. Interestingly, the most prominent examples of in silico drug screenings against COVID-19 seem to be based on molecular docking or molecular dynamic algorithms. Benefitting from the increased computational power, molecular docking algorithms are now suitable to screen giga-sized compound libraries against a single protein target. Such studies are testing tens of billions of compounds and are predicting a wide range of chemically diverse compounds^30,31^. Most screened libraries are focused on known drugs and their relatives, but other recent approaches are screening against libraries of natural compounds to increase the search space^23,32,33^.

Still, the major drawback of most in silico screenings is the lack of proper prediction validation resulting in only modest outcomes of huge screenings and no fast and global solution for the current pandemic ^34^.

**Figure 1:**
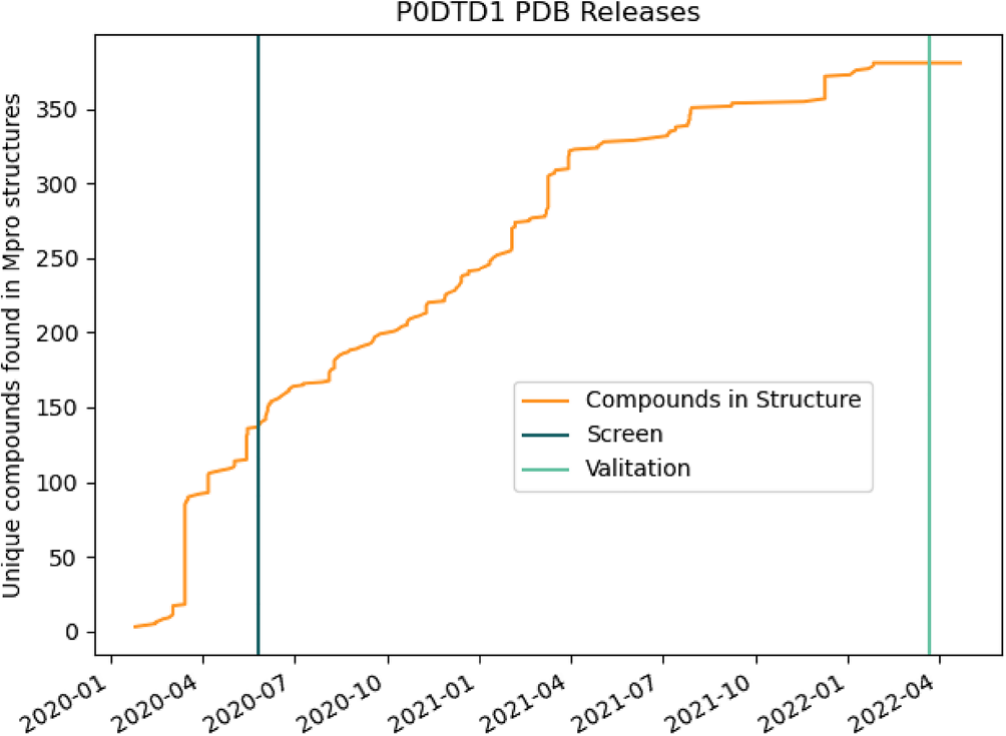
Unique compounds released in complex with M^pro^ in the PDB. Structures are searched by the UniProt ID P0DTD1 and filtered for interactions with M^pro^. Horizontal lines mark the days of M^pro^ inhibitor prediction and validation by data available in the PDB and ChEMBL.

**Figure 2:**
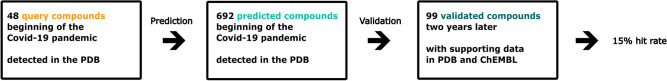
Graphical abstract. The pipeline consists of three major steps. First (left panel) 48 query complexes of M^pro^ with co-crystallized ligands are extracted from the PDB. Second (middle panel) the interaction patterns are transformed into one-dimensional fingerprints and screened against the full PDB database resulting in 692 predicted compounds. Third (right panel) these predictions are validated with publicly available data leading to 99 validated compounds that are associated with SARS-CoV-2. The validation implicates a hit rate of at least 15%.

By using a large amount of available data on the main protease of SARS-CoV-2, we want to address the above-mentioned problems. First, available M^pro^ compound complexes are extracted from the PDB and their binding patterns get analyzed by the Protein–Ligand Interaction Profiler (PLIP)^35^. Second, all protein–ligand complexes in the PDB are screened to detect similar binding patterns and predict potential inhibitors. Since we noted a drastic increase in publicly available data after the screen was done we decided to use this information for a further validation step. The data available in the PDB, before and after the screen, is depicted as a timeline in Fig. 1. Using this data and M^pro^ binding affinity values from ChEMBL we were able to semi-automatically validate the predictions. Following these steps, the predictions are not dependent on pure chemical properties and therefore expected to be very diverse, leading to potential interesting and never considered findings. The automated part of the validation does not require any wet lab work and only depends on publicly available data. The pipeline is summarized in Fig. 2.

This way, we were able to predict 692 unique potential M^pro^ inhibitors and validated 17% of the top 100 predictions retrospectively by publicly available data. The predictions cover a large chemical space and have great potential as lead compounds targeting M^pro^. Within the top 100 predictions, we identified 4 already FDA-approved drugs that are currently under investigation for the treatment of the COVID-19 disease. The analysis of specific binding patterns within all available M^pro^ compound complexes in the PDB confirmed the importance of potential drugs interacting with the catalytic dyad of M^pro^’s active site. We furthermore detected an interesting pattern of three almost perpendicular hydrogen bonds interacting with hydrogen donors of an oxyanion hole within the active side. Our work contributes to the scientific community's efforts to detect potential lead compounds for a given protein target in a fast and reliable way.

## Materials and methods

### Data extraction and prefiltering

A search of the PDB for M^pro^ on 21 March 2020 returned a set of 140 compounds found in complex with the protein. Those were filtered in two major steps. First generic and promiscuous compounds were filtered out using an in-house blacklist. Second, only those that bound the catalytic binding site of M^pro^ were considered, leaving only 48 compound- M^pro^ complexes. These 48 complexes served as input for an interaction-based screening using the PharmAI DiscoveryEngine (Version 2021.03, date 21 March 2021, https://www.pharm.ai). The small molecules in the PDB were set as target library for the predictions of the DiscoveryEngine.

### Interaction based screening

In these screening approaches the way a given ligand is interacting with a protein is extracted using software, such as the Protein–Ligand Interaction Profiler (PLIP)^36^ from three-dimensional complex data as provided by the protein database (PDB)^37^ as well as geometric matches of ligand and binding site. The interactions are afterward converted into one-dimensional vectors (interaction fingerprints). Such interaction fingerprints can be compared with others using comparison schemes, such as the Tanimoto similarity index or comparable techniques, to screen large databases. The screen returned 740 unique compounds. Similar screening strategies have been used in Salentin et al. 2017, Adasme et al. 2020, and Adasme et al. 2020^38–40^.

### Prediction evaluation and visualization

**Figure 3:**
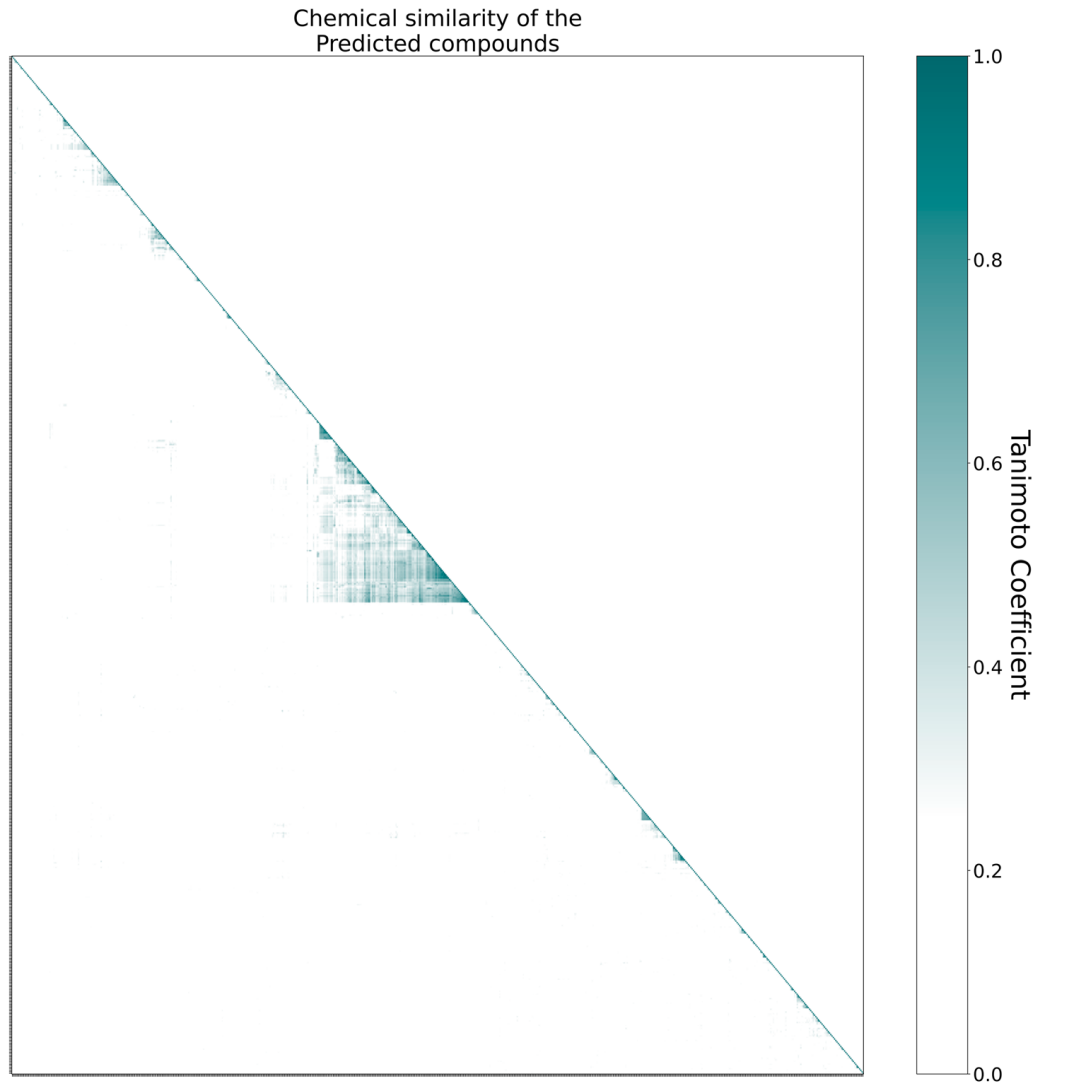
Chemical similarity heatmap of the 692 predicted compounds. Since the underlying matrix is symmetric, the upper triangle is not shown explicitly. The analysis reveals little redundancy and a broad spectrum of scaffolds. The big cluster (middle) consists of compounds similar to deoxyadenosine monophosphate which is a group known to bind M^pro^.

**Figure 4:**
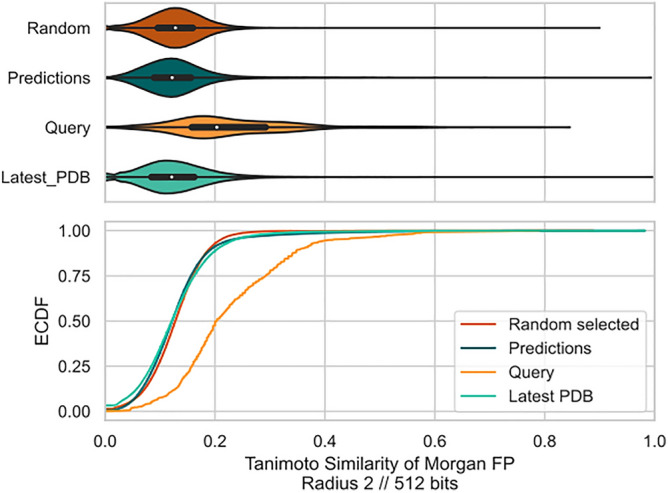
Pairwise chemical similarity of predicted, random, latest PDB, and query compounds. Top: violine plot. Bottom: empirical cumulative density function (ECDF) of similarities. Query compounds are more similar to each other than predictions, which are as similar to each other as a random set of compounds. This indicates that predictions substantially expand from the queries and cover a vast chemical space. 99% of random compounds have a similarity of less than 0.25 suggesting that 0.25 is a meaningful cut-off to consider compounds unrelated.

**Figure 5:**
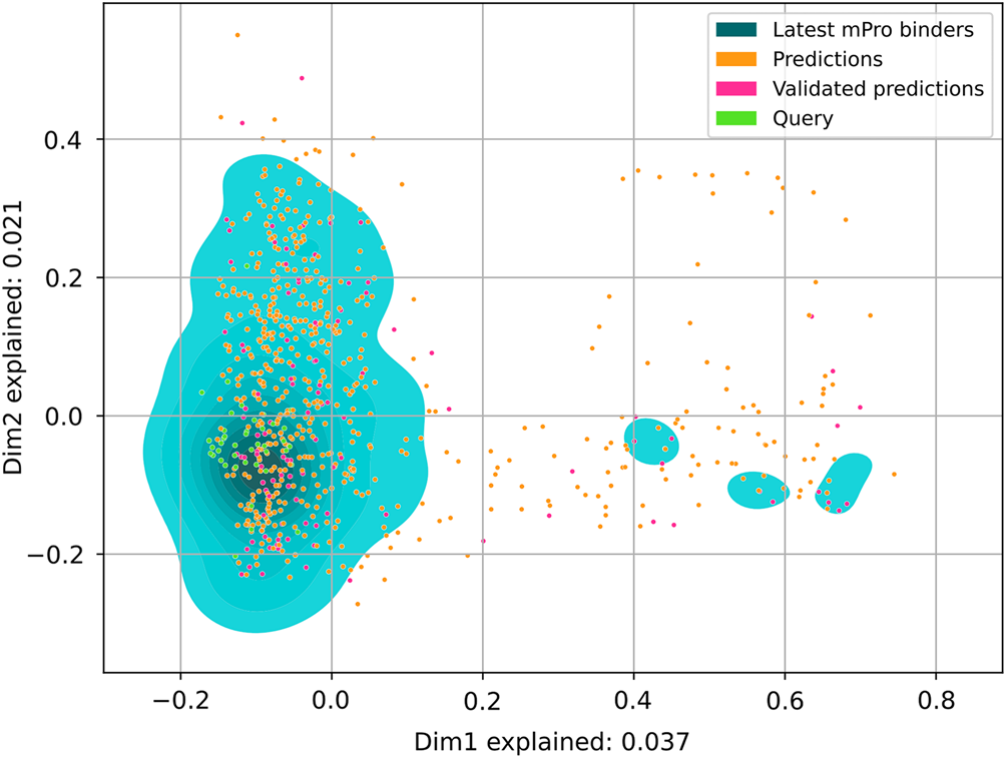
Multiple correspondence analysis (MCA) of predicted- (orange dots), validated- (magenta dots), query- (green dots), and known- (blue surface) M^pro^ binders. The axes of the MCA plot represent the dimensions of the data with the highest amount of explained variance. The analysis reveals that the predictions do cover a bigger chemical space than the known M^pro^ binders with structures available in the PDB.

48 predicted compounds, which were already in complex with M^pro^, were removed, resulting in 692 compounds. For these compounds, chemical fingerprints were computed using the Morgan fingerprint radius 2 and 512 bits^41^. The similarity of compounds was computed with the Tanimoto score, i.e. |A⋂B|/|A⋃B| where A and B are two vectors. A random set of 400 compounds was created to determine a cut-off for dissimilar compounds. 200 were selected from the total of all 35.153 compounds in PDB and 200 from the total of 2.157.379 compounds in ChEMBL (March 2022). There was no overlap between the two groups. Pairwise Tanimoto scores were computed, and their distribution indicated that 99% of pairs have a Tanimoto score of less than 0.25. Thus, 0.25 was used as a cut-off for dissimilar compounds. Compounds were clustered using hierarchical clustering with single linkage from scipy^42^. They were visualized as a heatmap (Fig. 3) with the cut-off of 0.25 to indicate dissimilar compounds. The multiple correspondence analysis and empirical cumulative density functions (Figs. 4,5) were computed using scipy^42^. Interactions of compounds to M^pro^ were extracted from PDB files using PLIP 2.2.0^35^ and visualized in Pymol. The hydrogen bond triple motif was flagged if PLIP identified a hydrogen bond in M^pro^ residue 143, 144, and 145.

To validate the results, we searched PDB and ChEMBL for compounds known to interact with M^pro^ to compare those with our predictions. PDB and ChEMBL were searched for the M^pro^ Uniprot ID P0DTD1 on 9 March 2022 and 22 March 2022, respectively. PDB returned 471 unique compounds and ChEMBL 7.221. All considered PDB structures are generated by X-Ray Diffraction with a resolution of at least 2.4 Å (see Suppl Suppl Appendix Table 1). All interactions in ChEMBL are from the same screen (CHEMBL4495582) and results are reported as M^pro^ inhibition percentage at 20 µM by FRET kind of response from peptide substrate^43^. Inhibitory activity was normalized to the one of Zn-Pyrithione as the positive control (100%) and DMSO as the negative control (0%). For the confirmation of valid hits, we assumed that reported compounds with values above 0% inhibition are at least weakly active.

## Results

### Structure-based drug screening for M^pro^ reveals 692 potential inhibitors

To identify repositioning candidates for the inhibition of M^pro^, predictions were provided by PharmAI (Dresden, Germany) as a result of an interaction-based screening. The screening revealed 692 potential M^pro^ inhibitors within the PDB. The predictions are further evaluated in three steps. First, their chemical properties are analyzed in terms of similarity to each other and known M^pro^ inhibitors. Here, we aim to find a heterogeneous set of predictions that cover chemical scaffolds beyond the already known ones with the potential of inhibiting M^pro^. Such novel predictions may function as the basis for further evaluation and drug design. Our analysis revealed that the predictions are indeed very heterogeneous and do cover a large chemical space. Second, the predictions are searched for already known binders that are found in the PDB or ChEMBL to get a first idea of the predictive performance of the screen and to include publicly available data. Furthermore, predictions of high importance as already FDA-approved drugs are checked for an association as a M^pro^ inhibitor or COVID-19 drug in general. By that, we can confirm that 17% of our top 100 predictions have evidence of binding M^pro^. Furthermore, 12 compounds are known to interact with other viral proteins of the replicase polyprotein 1ab, and we identify multiple FDA-approved drugs that are potential COVID-19 drug candidates. Third, we analyzed compound-M^pro^ binding patterns to detect potentially important binding modes and recognized a potentially important tripled hydrogen bond pattern.

### Predicted compounds are heterogeneous

The chemical properties of 692 predicted compounds were evaluated. To get a first impression of the chemical relations in the large prediction set, we created a heatmap of their pairwise chemical similarities. All similarities are calculated as the Tanimoto similarity score of Morgan chemical fingerprints which is a 2D descriptor (see “Methods”). Such an analysis gives insights into how chemically diverse a set of compounds is. For example, similar compounds would form one or a few big clusters in such a heatmap while dissimilar ones would form none or multiple very small clusters. Ideally, the predicted compounds consist of new scaffolds covering a large chemical space. An outcome like this can give new insights into chemical species that should be considered as the groundwork for further drug design approaches.

Comparing chemical species is a challenging task and is usually done by transferring string representations of the compound into vector representations that can be compared by metrics such as the Tanimoto similarity index. Since all of such approaches come with their own benefits and drawbacks, we benchmarked the used combination of the Morgan fingerprint with radius 2 and 512-bit representation combined with the Tanimoto similarity index. Evaluating the similarity of 400 randomly selected compounds (Fig. 4) revealed that 99% have a similarity of less than 25% suggesting that this is a meaningful cut-off to consider compounds related/unrelated.

The heatmap analysis (Fig. 3) revealed that in all but one case only small clusters are formed. Similarities below 25% are whited out since those compounds can be treated as unrelated. The big cluster (118 out of 692 compounds) consists primarily of deoxyadenosine monophosphate derivatives. This result is not surprising since the already FDA-tested drug Remdesivir and its active metabolite GS-441524 are adenosine derivatives as well. These types of inhibitors are already shown to successfully inhibit viral replication. Other derivatives e.g. Cordycepin yield M^pro^ binding affinity^44–46^. This gives further support for the predicted compounds. Nonetheless, the majority of compounds are unrelated, suggesting that the predictions are indeed chemically diverse.

### How do the predictions relate to known inhibitors?

In general, predictions that cover a large chemical space are more likely to reveal interesting and novel scaffolds that can even be more important than a high hit rate^47^. Figure 5 shows the multiple correspondence analysis (MCA) applied to the chemical Morgan fingerprints of our predictions and all compounds with structures available in the PDB where they are in complex with M^pro^. Given in blue is the kernel density estimate (KDE), i.e. the probability distribution, of the PDB M^pro^ binders, orange dots mark the predictions, green dots mark query compounds, and magenta dots mark validated predictions. The analysis implies that the predictions fill a larger chemical space compared to the known binders and query compounds. Most of them are found in high-density regions of the known binders, which supports the overall approach since they do not form a whole new chemical space. The same holds true for validated predictions. However, we indeed identified compounds that are beyond the chemical space of known binders.

To access the heterogeneity of the predicted compounds even further we computed the pairwise similarity of 400 randomly selected compounds (200 ChEMBL, 200 PDB). The result is shown in the top panel of Fig. 4. Only the set of query compounds seems to show some degree of homogeneity with a mean chemical similarity of 0.23, which is still below our prior defined threshold. The randomly selected compounds, predictions, and known M^pro^ PDB binders have mean similarities around 0.125.

In summary, the predicted compounds seem to be as heterogeneous as known and tested M^pro^ binders while containing new scaffolds that may contribute to future efforts in developing a M^pro^-specific anti-COVID-19 drug.

### The validation with publicly available data revealed a hit rate of 17%

After evaluating the predictions based on their chemical features, we aimed to validate them. Doing this for more than 600 compounds in vitro is a huge effort and we, therefore, make use of the astonishing amount of publicly available data on SARS-CoV-2. Here we have three principal approaches: first we extracted all compounds that are found co-crystallized with SARS-CoV-2 viral proteins in the PDB. Figure 1 gives an overview of structures published with the UniProt ID P0DTD1 that are co-crystallized with M^pro^. Second, we searched ChEMBL for released affinity values of experiments with the target M^pro^ (CHEMBL4523582). For this section of the analysis, ChEMBL was selected due to its accessibility and the thorough curation of the provided data. Lastly, we evaluated FDA-approved predicted drugs by literature search.

Compounds are considered to be validated in PDB if a structure is available with a predicted compound in complex with the protein target M^pro^. In addition to these four compounds, we identified another 12 which are found in complex with other proteins of the replicase polyprotein 1ab (see Suppl Appendix Table 1). After the screening was performed in 2020, 420 new structures of M^pro^ were released, which serve as a basis for this part of the validation.

Since PDB is very limited due to its small number of available compounds (34,204) we investigated our results against ChEMBL as well. ChEMBL was searched for activity evidence on the reported predictions and M^pro^. Interestingly, to date, there is only data of a single high throughput screening on M^pro^ available in ChEMBL. For a total of 100 compounds, there is activity evidence, however only inhibition percentage values at 20 µM compound concentration are provided. Out of those 100 compounds, 76 show relative inhibition of > 10%, 30 more than 20%, and 11 more than 30%. It is therefore hard to judge if those are strong (nanomolar binders) or compounds that are only weakly interacting with M^pro^. Detailed information on the predictions and validation data can be found in Suppl Appendix Table 1.

Nonetheless, the compounds are active which gives evidence beyond estimated interaction patterns, and even non-nanomolar binders are potential foundations for further drug optimization. Strangely, there is hardly any overlap between compounds found in ChEMBL and PDB even though M^pro^ is currently one of the most studied proteins. Among all 99 validated compounds, only 7 are found to have activity values reported in ChEMBL and a structure in complex with an viral protein available in the PDB. The lack of more activity data in ChEMBL can be attributed to the fact that ChEMBL has a very strict and standardized review procedure.

In summary, the performed in silico screening has an in vitro hit rate of 15% within all 692 predicted compounds and a hit rate of 17% within the top 100 predictions, ranked by p-values (Table 1). Thus, there is substantial evidence that the predictions are indeed valid drug candidates against SARS-CoV-2.

**Table 1:** Validation of predicted compounds.

	PDB	ChEMBL	Both
Top 100	2 (2%)	15 (15%)	17 (17%)
All 692	4 (0.5%)	100 (14%)	99 (15%)

The top predictions are highly enriched in independently validated M^pro^ binders. Validation is done by evaluating with identical compounds that show inhibitory activity in ChEMBL or found in complex with M^pro^ in the PDB. Given values for PDB and ChEMBL validation do not consider any overlap.

### Further evaluation supports prior findings on four FDA-approved drugs

Next, we want to get a deeper understanding of these predictions. We assess them by the interaction motifs present in the query structures and predictions, by highlighting the two most strongly validated predictions with evidence in both ChEMBL and PDB, and third by evaluating predictions of FDA-approved drugs with literature or clinical trial evidence as anti-COVID drugs.

**Figure 6:**
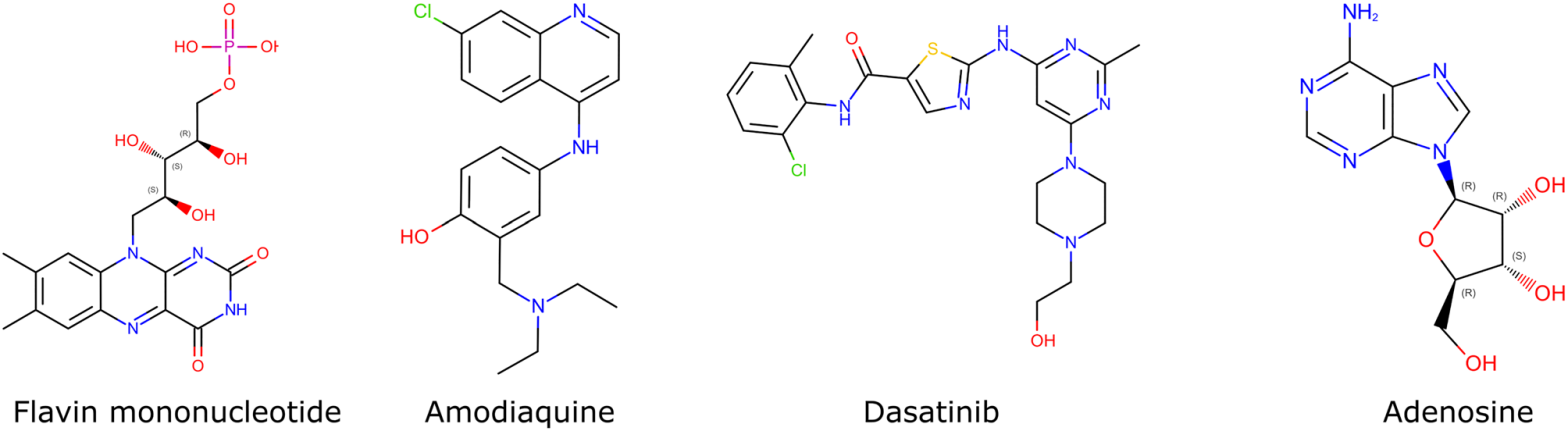
Structures of four FDA-approved predictions with evidence on COVID-19. All are part of the top 100 predictions.

Among the top 100 predictions, four are approved for use in humans by the U.S. food and drug administration (FDA), which are Flavin mononucleotide, Amodiaquine, Dasatinib, and Adenosine (Fig. 6). Flavin mononucleotide (FMN) is an orange-red food color additive and is predicted in complex with UbiX from the psychrophilic bacterium colwellia psychrerythraea (PDB:4REH)^48^. In 2022, Akasov et al. gave evidence about the usage of riboflavin supplementation to decrease inflammation in COVID-19 patients^49^. The malaria drug Amodiaquine is predicted in complex with human histamine N-methyltransferase (HNMT), which is a histamine-inactivating enzyme (PDB:2AOU)^50^. Amodiaquine was found to block SARS-CoV-2 infection with an EC50 value of 0.13 μM and was already proposed as a potential candidate against the early phases of the infection^51^. It was furthermore predicted to be a fruitful inhibitor of M^pro^ in a molecular docking study performed by Hagar et al. in 2020^52^. Dasatinib is a known tyrosine kinase inhibiting drug approved for use in patients with chronic myelogenous leukemia and is predicted in complex with the human SH2-kinase domain (PDB:4XEY)^53^. In a clinical case, Dasatinib (100 mg/day) reduced fever, and a duplicate swab test came out negative two weeks later^54^. However, it was unclear with which protein target the drug was interacting^55^. Furthermore, Dasatinib in combination with Quercetin reduces lung inflammation in SARS-CoV-2 infected hamsters and mice^26^ and is now in phase two of clinical trials as an anti-inflammatory drug in patients with moderate and severe COVID-19 (https://clinicaltrials.gov/ct2/show/NCT04830735). Adenosine is an organic body-own compound and showed promising anti-inflammatory effects in COVID-19 patients when inhaled^56,57^. In addition, the adenosine analog cordycepin was found to potently inhibit viral replication of resistant SARS-CoV-2 strains with an in vitro EC50 value of only 2 µM.

Despite the existing evidence of viral inhibition, the specific mechanisms of action for all four molecules remain unclear, necessitating the need for an in vitro demonstration of M^pro^ inhibition.

### The evaluation of recently released PDB M^pro^ structures reveals a common interaction pattern

**Figure 7:**
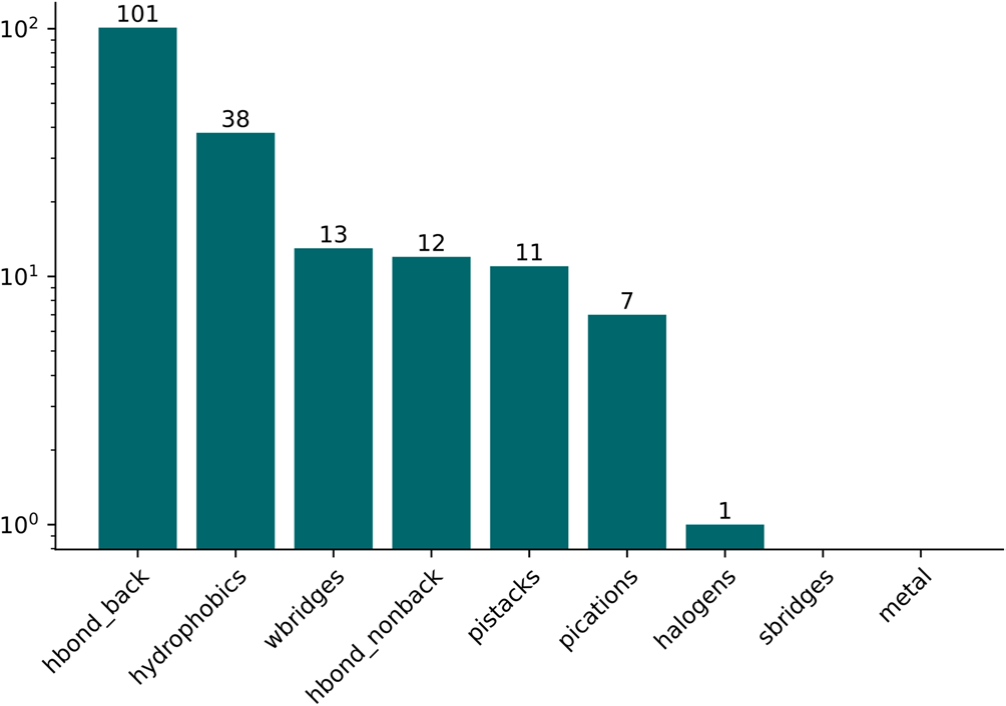
Interaction types present in 48 query compounds.

In addition to using recently published data on M^pro^ to validate inhibitor predictions, the data was used to get supplemental insights on the binding mode. Starting from the most high-level perspective on the interactions we calculated the frequency of each main interaction type. It was previously shown that the most frequent interaction type in the PDB are hydrophobic interactions^58^. As depicted in Fig. 7, the most frequent interaction types among M^pro^ binders are hydrogen bonds followed by hydrophobic interactions and water bridges. There is some specificity in the compound M^pro^ interactions compared to what is generally present in the PDB.

Not surprisingly, a total of 121 out of 471 unique compounds are interacting with one or both amino acids composing the catalytic dyad. Notably, the His41 residue exhibited a diverse range of interactions, with 39 pi-stacking interactions, and 23 hydrophobic interactions dominating the scene. Additionally, hydrogen bonds (8), pi-cation interactions (7), water bridges (4), salt bridges (2), and even halogen bonds (1) were also detected, providing a complex and intriguing picture of the binding interactions at play. Interestingly, Cys145 displayed a clear preference for hydrogen bonding interactions, with a remarkable 73 compounds interacting via this mode. Other interaction types, such as water bridges (2) and hydrophobic interactions (1), were also observed, hinting at the complexity and diversity of the catalytic dyad's interactions with ligands.

Further investigation on M^pro^ binding modes results in the identification of a potentially interesting triplet hydrogen bond pattern present in 35 out of 471 structures.

**Figure 8:**
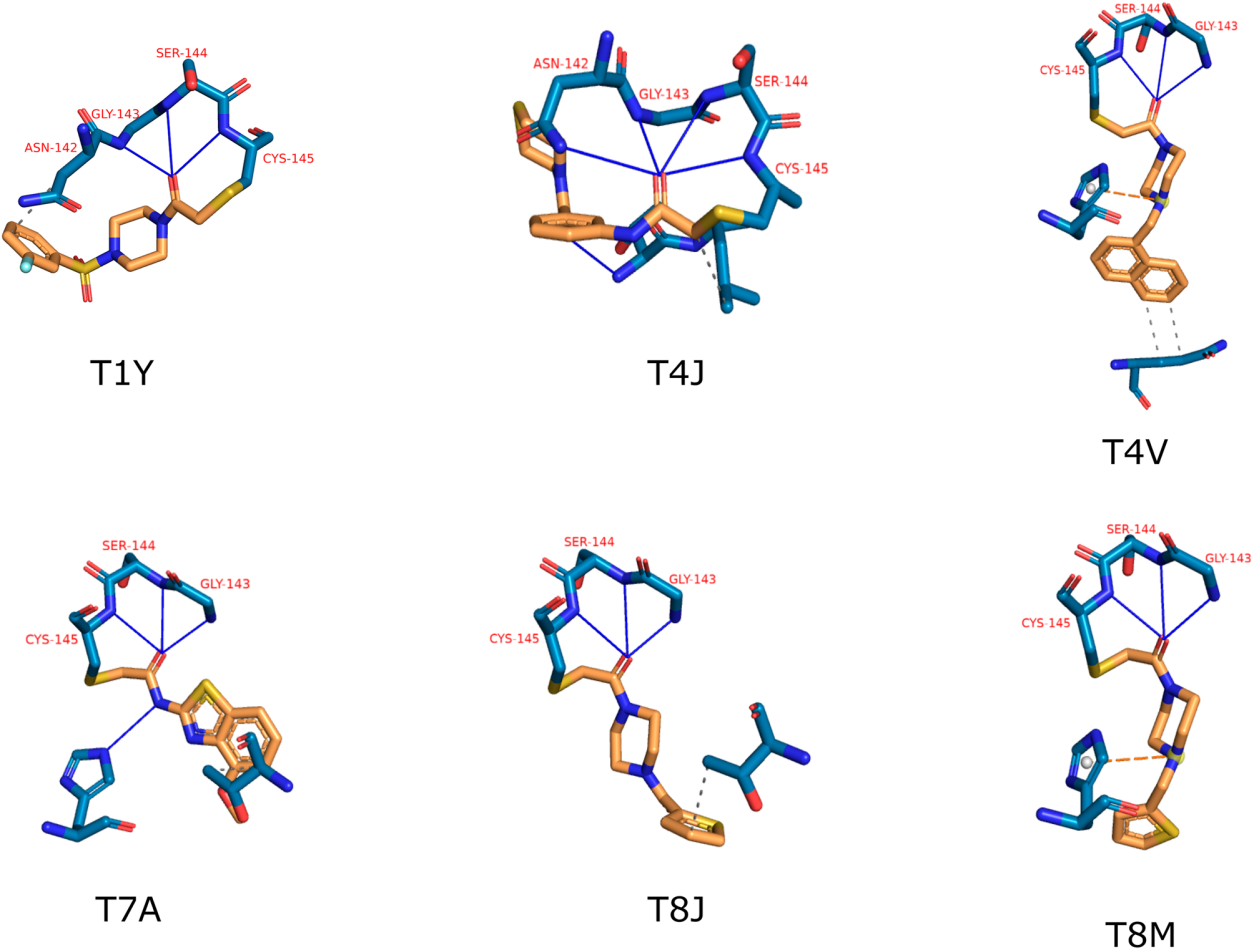
Protein (blue) compound (orange) interactions of selected compounds. Blue lines mark hydrogen bonds, orange dashed lines mark pi-cation interactions, and dashed grey lines mark hydrophobic interactions. The three-letter codes refer to PDB chemical ids. Residues are indicated in red. A specific motif of three nearly perpendicular hydrogen bonds is present in six of the 48 query compounds.

In Fig. 8, we showcased six examples that were used as input for the compound predictions. The compounds form three hydrogen bonds with the residues Gly143, Ser144, and Cys145. This finding is in agreement with what is reported by Douangamath et al. in 2020^25^. Here they found, that co-crystallized electrophilic ligands tend to form either two or three hydrogen bonds with Gly 143, Ser 144, or Cys 145. A similar pattern was previously reported by Zhang et al. in 2020^10^ and is an addition to the importance of interactions with the catalytic dyad composed of His41 and Cys145. This triplet interaction is of major importance for the protease function since Gly143, Ser144, or Cys145 do function as hydrogen bonding donors of the oxyanion hole present in M^pro^’s active side^59^. Therefore, we expect compounds that are able to dive deeply into the pocket and form interactions with those residues will efficiently inhibit the protease.

**Figure 9:**
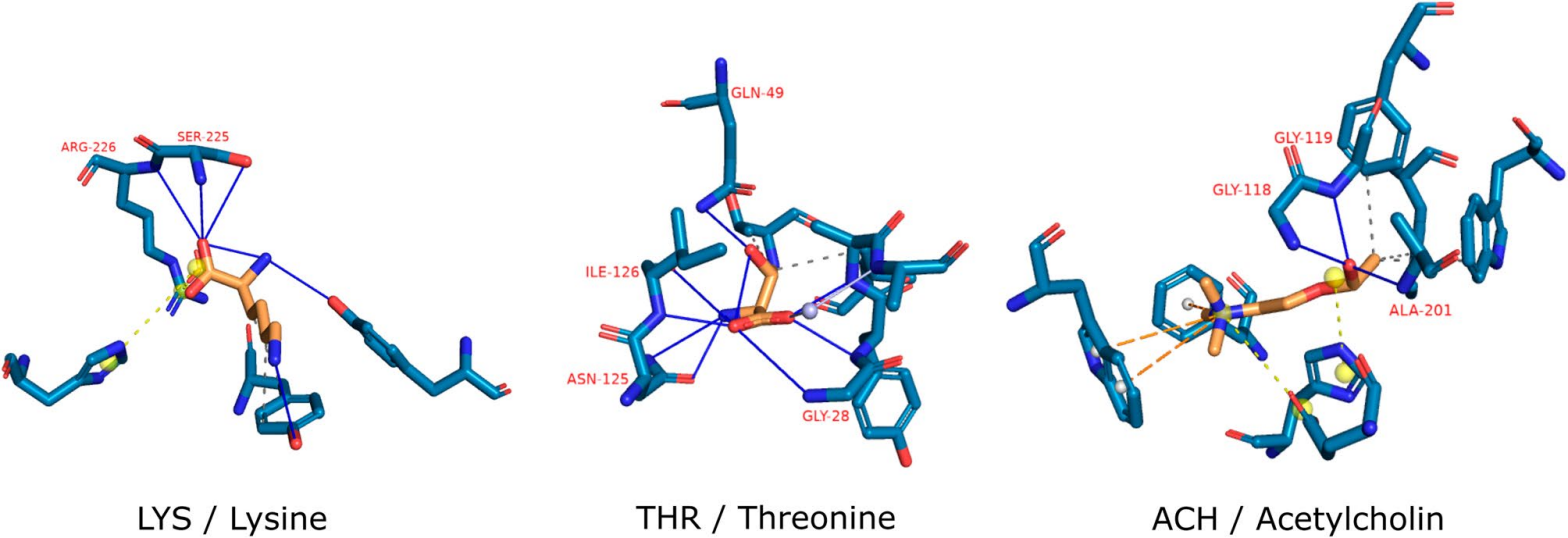
Protein (blue) compound (orange) interactions of selected compounds. Blue lines mark hydrogen bonds, dashed orange lines mark pi-cation interactions, dashed yellow lines mark salt bridges, and dashed grey lines mark hydrophobic interactions. Residues are indicated in red. The three-letter codes refer to PDB chemical ids. Interacting proteins from left to right are: SET domain lysine methyltransferases (UniProt: Q43088), aspartokinase (UniProt: P9WPX3), and acetylcholinesterase (UniProt: P04052). The triple hydrogen motif is present in multiple predictions as well as in 35 out of 471 M^pro^ complexes in PDB.

Turning the attention to our drug candidates, we identified a very similar pattern in three predicted structures (Fig. 9), all of which are complexes with FDA-approved drugs. These cherry-picked examples show the opportunity of detecting similar patterns in different proteins by interaction-based prediction methods.

## Discussion

The current COVID-19 pandemic exemplifies that fast-spreading diseases are a serious threat to modern society. By structure-based drug repurposing, we can predict a chemically diverse set of potential lead compounds against the main protease of SARS-CoV-2 with a success rate of 17%. Within the set of validated compounds, we identified several FDA-approved drugs, of which some are currently tested in clinical trials against SARS-CoV-2. Furthermore, we exploited the binding mode of known M^pro^ inhibitors and revealed the potential importance of a triplet hydrogen bond pattern for the protein–compound interaction.

Performing in silico drug screenings is a challenging task and comes with its own benefits and drawbacks. In contrast to wet lab studies, they are rather inexpensive, safe, and cheap. However, the result is only a prediction that requires experimental validation. Several researchers took the challenge of the COVID-19 pandemic and applied their very own algorithms aiming to predict fruitful drug candidates for multiple viral targets. Nonetheless, several of these studies do lack any kind of validation leaving the reader of such articles to judge themselves on how trustworthy the results in general are. Others created a full pipeline starting from in silico predictions which are then meticulously experimentally tested on important parameters, such as binding, cytotoxicity, metabolic stability, or oral receptivity ^9,20^.

Drug repurposing already led to some successes in the context of the COVID-19 pandemic. Owen et al. proved in 2021 that by chemically modifying and improving a predicted lead compound an efficient drug against a given disease can be developed^20^. Their drug Nirmatrelvir is now conditionally approved in the EU and US. Even though this is a great success, their lead compound was already predicted as a potential drug against the SARS-CoV-1 outbreak in 2002. Still, it shows that experts in the field can rapidly develop potent drugs in a relatively short period of time when starting from an appropriate lead molecule. Following this assumption, we aimed to predict a chemically diverse set of potential M^pro^ inhibitors with our interaction-based approach. In doing so, the chances to detect so far unknown but potentially very important compound scaffolds are increased, giving more value to the predictions. We are able to show that the predictions are not only little redundant but furthermore cover a large chemical space including so far untested scaffolds. This is especially important considering that the query compounds used as the input for the prediction are far more homogeneous compared to the predictions and validated predictions. The same holds true for validated predictions, suggesting, that the scientific community is already heavily increasing the diversity of tested small molecules against COVID-19. Moreover, it is a proof of concept, that chemically diverse small molecules can still be effective as inhibitors for the same protein target.

This opens the gates for further developments based on our predictions. The most limiting factor is the availability of compounds in the PDB that are the only ones considered in the screen due to the requirement of protein–compound complexes as input for the algorithm.

Furthermore, the herein presented method aims to predict small molecules targeting a specific active site and does not allow for reliable predictions on molecules targeting e.g. allosteric binding sites. However, these can be included in a screen if interaction data is available in the PDB. By using publicly available data, we have created an intermediate approach that yields more trustworthy results than comparable in silico approaches but is not as powerful as those who considered experimental validation. With a hit rate of at least 17% within the top 100 predictions and 15% overall, the algorithm performance is substantial compared to similar approaches^31^.

The evaluation of FDA-approved drugs within the predictions revealed the potential of the method to generate new hypotheses on drug mechanisms. All compounds are predicted to inhibit the main proteases of the Sars-CoV-2 virus and should therefore prevent viral replication. Through literature research, we identified articles on four FDA-approved drugs, showing beneficial effects in COVID-19 patients, that are within our top 100 predictions, and none of those reported any drug mechanism. The drugs Riboflavin, Amodiaquine, Dasatinib, and Adenosine have shown anti-inflammatory effects in COVID-19 patients or in-cell antiviral activity^49,51,55–57^. This raises the question of whether reduced viral replication mediated by the inhibition of M^pro^ as predicted by us is responsible for the reduced inflammation.

Ascorbic acid on the other hand is one of our validated and FDA-approved predictions but there is evidence that it is not applicable as a COVID-19 drug due to its inefficiency in infected patients^60,61^. This exemplifies the limitations of the approach. Even if a drug does bind and eventually inhibits a target protein, there is no guarantee that it could function as a drug. Factors such as cell permeability, half-time, or other mechanisms can counteract the inhibitory properties of a compound. That can not be tested in a pure in silico fashion and does require wet lab work.

Anyway, the elephant in the room here is the other 82% of the predictions without validation. So far, there is no evidence of these compounds interacting with M^pro^ found in the PDB or ChEMBL. Therefore, this set of compounds may contain fruitful new lead scaffolds and their identification does require further experimental validation and evaluation.

Supplementary analysis on interaction patterns of recently released M^pro^-compound complexes reveals a triplet hydrogen bond that could explain stable interactions and efficient inhibition. Compounds with such a binding mode do interact with all neighboring residues of the oxyanion hole (Gly143, Ser144, Cys145) and are therefore blocking its catalytic function. Since only 13% of the M^pro^ complexes in the PDB do show such a pattern, further investigations are required to test if those do have lower binding energy as we expect. Still, similar patterns are reported by different research groups highlighting the importance of further investigations regarding its importance on M^pro^ inhibition.

## Conclusion

With our work on SARS-CoV-2, we can show that our interaction-based prediction method has great potential to predict a diverse set of potential lead compounds for a given protein target. Starting from a relatively homogeneous and small set of compound fragments bound to the main proteases of SARS-CoV-2, we predicted a chemically diverse set of potential inhibitors. Overall, we produced lead compound predictions at a very high hit rate by our interaction-based approach and were able to perform a first validation without the requirement of additional wet-lab work.

In this work, we benefited from the data-rich situation, but the method is applicable as long as there are complexes of the target protein bound to a compound available in the PDB. That way, we can provide a foundation for further lead optimization for lots of disease-associated proteins enhancing the drug development process.

## Supplementary Information

Supplementary Table 1.

## Data Availability

The interaction data used as input for the predictions can be found in Suppl Appendix Table one column “Query PDB ID:Chemical ID”. The corresponding PDB files are publicly available from the PDB (https://www.rcsb.org). All resulting predictions can be found in Suppl Appendix Table one column “Hit PDB ID:Chemical ID”.
